# Low EGFR/MET ratio is associated with resistance to EGFR inhibitors in non-small cell lung cancer

**DOI:** 10.18632/oncotarget.5131

**Published:** 2015-09-03

**Authors:** Silvia Park, Emma Langley, Jong-Mu Sun, Steve Lockton, Jin Seok Ahn, Anjali Jain, Keunchil Park, Sharat Singh, Phillip Kim, Myung-Ju Ahn

**Affiliations:** ^1^ Division of Hematology-Oncology, Department of Medicine, Samsung Medical Center, Sungkyunkwan University School of Medicine, Seoul, Korea; ^2^ Prometheus Laboratories Inc, A Nestlé Health Science Company, Department of Research and Development, San Diego, CA, USA

**Keywords:** NSCLC, EGFR TKI, PFS, EGFR/MET ratio, HER3

## Abstract

**Purpose:**

Although activating mutations in the epidermal growth factor receptor (*EGFR*) gene are predictive markers for response to EGFR inhibitors, 30–40% of *EGFR*-mutant non-small cell lung cancer (NSCLC) patients are *de novo* non-responders. Hence, we sought to explore additional biomarkers of response.

**Methods:**

We conducted a prospective pilot study to characterize the expression and/or activation of key receptor tyrosine kinases (RTKs) in stage IIIB-IV NSCLC tumors. A total of 37 patients were enrolled and 34 underwent EGFR inhibitor treatment.

**Results:**

As expected, patients bearing activating *EGFR* mutations showed increased progression free survival (PFS) compared to patients with wild-type *EGFR* status (9.3 vs 1.4 months, *p* = 0.0629). Analysis of baseline tumor RTK profiles revealed that, regardless of *EGFR* mutation status, higher levels of EGFR relative to MET correlated with longer PFS. At multiple EGFR/MET ratio cut-offs, including 1, 2 and 3, median PFS according to below vs. above cut-offs were 0.4 vs. 6.1 (*p* = 0.0001), 0.5 vs. 9.3 (*p* = 0.0006) and 1.0 vs. 11.2 months (*p* = 0.0008), respectively.

**Conclusion:**

The EGFR/MET ratio measured in tumors at baseline may help identify NSCLC patients most likely to benefit from prolonged PFS when treated with EGFR inhibitors.

## INTRODUCTION

Lung cancer is the most prevalent type of cancer, and is the leading cause of cancer death worldwide [1, 2]. The overall 5 year survival rate is low at 15% with over half the patients dying within 1 year of diagnosis. Non-small cell lung cancer (NSCLC) accounts for 80 to 85% of lung cancer and most patients are diagnosed at an advanced stage. Current treatment options for NSCLC typically include a combination of surgical resection, platinum-based doublet chemotherapy, radiation therapy and/or targeted therapy [3, 4].

A greater understanding of the molecular mechanisms driving lung carcinogenesis prompted the development of targeted therapies for the treatment of NSCLC in recent years [5–7]. These targeted therapies are designed to antagonize oncogenic ‘driver’ mutations and/or genetic abnormalities that confer a growth and survival advantage to lung cancer cells. In the past decade, many novel drugs, which include monoclonal antibodies and small molecule inhibitors, have been evaluated in numerous clinical trials and several are currently approved for NSCLC treatment [8–13].

The epidermal growth factor receptor (EGFR) is a receptor tyrosine kinase (RTK) that induces cellular proliferation, differentiation, migration, survival and angiogenesis when activated by the binding of one of its ligands [14–16]. Abnormally elevated EGFR signaling is associated with many common human solid tumors, including lung cancer. In fact, the *EGFR* gene is frequently mutated in 10–15% of Caucasian and 30–40% of Asian NSCLC patients [17]. Reversible small molecule inhibitors of EGFR, such as gefitinib and erlotinib, exert anti-tumor activity in heavily pretreated NSCLC patients with few side effects and were initially approved for 2^nd^/3^rd^ line settings [18–21]. Furthermore, in 2013, erlotinib and afatinib, an irreversible EGFR family inhibitor, were approved for 1^st^ line therapy in NSCLC patients bearing activating *EGFR* mutations [10, 22, 23]. The most prevalent activating mutations, exon 19 deletion or L858R substitution, occur in the kinase domain and are mainly observed among patients with adenocarcinoma histology, never smokers, and East-Asian ethnicity [24–26].

The first randomized phase III trial comparing gefitinib with first-line carboplatin and paclitaxel in East-Asian never-smokers or former light smokers with lung adenocarcinoma demonstrated superiority of gefitinib in terms of response rate (RR) and progression free survival (PFS) [27]. In this study, subgroup analysis according to *EGFR* mutation status showed significantly higher RR and prolonged PFS in *EGFR*-mutant patients only when treated with gefitinib. In contrast, *EGFR* wild-type patients did worse with gefitinib compared to those treated with combination chemotherapy. Several subsequent randomized phase III studies conducted both in Asian and Western countries consistently demonstrated similar results. Hence, *EGFR* activating mutations are predictive biomarkers of high RR and prolonged PFS for EGFR tyrosine kinase inhibitor (TKI) therapy in NSCLC [22, 23, 25, 28–30]. The median PFS in *EGFR*-mutant patients treated with EGFR TKIs is typically between 9 and 11 months. The response duration, however, spans from months to years among individual patients, suggesting heterogeneity within this seemingly well-defined population. Although preexistence of the *EGFR* T790M gatekeeper mutation is considered one of the explanations, the exact mechanisms for primary resistance or very short duration of response to EGFR TKIs among *EGFR*-mutant patients have not been fully explored yet [31–33]. In addition to EGFR activity, alternate signaling pathways may be stimulated in such tumors, underscoring the need for a more comprehensive analysis of tumor pathway circuitries in each patient.

While characterizing tumor signaling pathways is highly desirable, it is challenging in the clinical setting where tissue availability is mostly limited. Thus, the use of methods that simultaneously evaluate the expression and activation of key components in limited amount of specimen is required. Using a highly sensitive and specific multiplexed immunoassay, we report here RTK pathway characterization of tumor cells from a cohort of NSCLC patients. We find that higher levels of EGFR relative to MET, another RTK with oncogenic properties in lung cancer, correlate with prolonged PFS to EGFR TKIs. Thus, the quantitative relationships between RTKs with compensatory functions, may have potential predictive value in the clinic and help guide therapeutic choices.

## RESULTS

### Patient characteristics

A total of 37 NSCLC patients were enrolled in the trial, and the baseline characteristics of this cohort are listed in Table 1. The median age was 57 years (range, 39–75 years) and both genders were almost equally represented (female [*n* = 20, 54.1%] and male [*n* = 17, 45.9%]). With the exception of one case, all cancers were of adenocarcinoma histology (*n* = 36, 97.3%). *EGFR* mutation testing revealed that 6 patients (16.2%) lacked information on mutation status, 9 patients had wild-type *EGFR* status (24.3%), and 22 patients carried *EGFR* mutations (59.4%). Among the latter group, activating mutations including exon 19 deletion and missense mutation at exon 21 (L858R) were present in 9 (24.3%) and 10 patients (27.0%) respectively; the remaining 3 patients (8.1%) had non-activating mutations. Gefitinib, erlotinib, and afatinib were used in 19 (51.4%), 14 (37.8%), and 1 (2.7%) patients, encompassing 1^st^ line (*n* = 6, 17.6%), 2^nd^ line (*n* = 19, 55.9%), 3^rd^ line (*n* = 8, 23.5%) and 4^th^ line therapy (*n* = 1, 2.9%), respectively.

**Table 1:** Patients characteristics (*n* = 37)

Patients characteristics		Number (%)
Median age	years (range)	57 (39–75)
Gender	Male	17 (45.9%)
	Female	20 (54.1%)
Pathology	Adenocarcinoma	36 (97.3%)
	Non-small cell carcinoma	1 (2.7%)
Epidermal growth factor receptor (*EGFR*) mutation	No mutation	9 (24.3%)
	Mutation	
	deletion 19	9 (24.3%)
	missense mutation 21 (L858R)	10 (27.0%)
	other mutation	3 (8.1%)
	Not assessed	6 (16.2%)
Tyrosine kinase inhibitor (TKI) use	Gefitinib	19 (51.4%)
	Erlotinib	14 (37.8%)
	Afatinib	1 (2.7%)
	No use	3 (8.1%)
TKI line (among 34 patients with TKI use)	1^st^ line	6 (17.6%)
	2^nd^ line	19 (55.9%)
	3^rd^ line	8 (23.5%)
	4^th^ line	1 (2.9%)
Best response to TKI (among 34 patients with TKI use)	PR	17 (55.0%)
	SD	6 (17.6%)
	PD	8 (23.5%)
	Not assessed	3 (8.8%)

PR: partial response; SD: stable disease; PD: progressive disease;

### Clinical outcomes to EGFR TKIs

Out of 37 patients, 3 patients did not use any TKIs after enrollment, and were excluded from subsequent analyses. *EGFR*-activating mutations were present in 19/34 (55.9%) patients with TKI use and equally distributed in each gender. As summarized in Table 2, a high proportion of *EGFR*-activating mutation positive patients responded to EGFR TKIs (15 out of 17 patients with evaluable responses, 88.2%). However, 5 out of 9 evaluable patients (55.6%) with wild-type or non-activating mutation *EGFR* status also showed evidence of treatment response (partial response [PR] or stable disease [SD]). Out of 8 non-responders (progressive disease [PD]) to EGFR-TKIs, 2 (25%) carried *EGFR*-activating mutations. While patients with *EGFR*-activating mutations showed increased median PFS over patients with wild-type *EGFR* status (9.3 months vs 1.4 months, *p* = 0.0629; Supplementary Fig. S1), statistical significance was not reached most likely due to small size of clinical cohort. Of note, however, an *EGFR* wild-type patient (006-004) experienced a remarkable clinical response with PFS of 23.4 months. On the contrary, 2 patients carrying an *EGFR*-activating mutation (006-032 and 006-044) appeared to be primary non-responders and did not demonstrate any clinical benefit despite their genotype.

**Table 2:** Clinical outcomes to EGFR TKI (*n* = 34)

Patient Number	Sex	*EGFR* activating mutation	TKIs	TKI line	PFS with TKI (month)	Best response to TKI	Patient Number	Sex	*EGFR* activating mutation	TKIs	TKI line	PFS with TKI (month)	Best response to TKI
006-002	F	Positive	Gefitinib	2	26.2	PR	006-026	F	Positive	Gefitinib	3	11.2	PR
006-003	M	Positive	Erlotinib	3	1.1	SD	006-027	M	Positive	Erlotinib	3	NA	NA
006-004	F	Negative	Gefitinib	2	23.4	PR	006-028	F	Positive	Erlotinib	1	NA	NA
006-005	M	NA	Erlotinib	2	0.2	PD	006-029	F	Positive	Gefitinib	2	9.3	PR
006-006	F	Negative	Gefitinib	1	0.9	PD	006-030	F	NA	Gefitinib	2	2.1	SD
006-008	M	Negative	Erlotinib	1	6.1	PR	006-031	F	Positive	Erlotinib	2	10.8	PR
006-009	M	NA	Gefitinib	2	1.8	PD	006-032	M	Positive	Erlotinib	2	0.5	PD
006-010	M	NA	Gefitinib	2	5.1	PR	006-034	F	Negative	Gefitinib	2	2.2	SD
006-012	F	Negative	Gefitinib	2	5.4	SD	006-035	F	Negative	Erlotinib	3	0.6	PD
006-013	F	Positive	Gefitinib	2	8.6	PR	006-037	F	Positive	Gefitinib	2	5.1	PR
006-014	F	Negative	Gefitinib	2	1.4	PD	006-038	M	Negative	Erlotinib	4	NA	NA
006-017	M	NA	Erlotinib	3	27.1	PR	006-039	F	Negative	Gefitinib	3	0.4	SD
006-018	F	Negative	Gefitinib	1	0.5	PD	006-040	M	Positive	Erlotinib	2	14.4	PR
006-019	M	Positive	Gefitinib	2	4	PR	006-041	F	Positive	Gefitinib	1	2.4	SD
006-021	F	Positive	Gefitinib	2	25.3	PR	006-042	M	Positive	Erlotinib	2	1.8	PR
006-023	M	Positive	Gefitinib	3	17.2	PR	006-043	M	Positive	Erlotinib	2	11.5	PR
006-025	F	Positive	Erlotinib	3	13.7	PR	006-044	M	Positive	Afatinib	1	1	PD

PR: partial response; SD: stable disease; PD: progressive disease; NA: not assessed.

### Mutation evaluation is insufficient to capture heterogeneity in NSCLC and to predict response to EGFR TKI treatments

**Figure 1:**
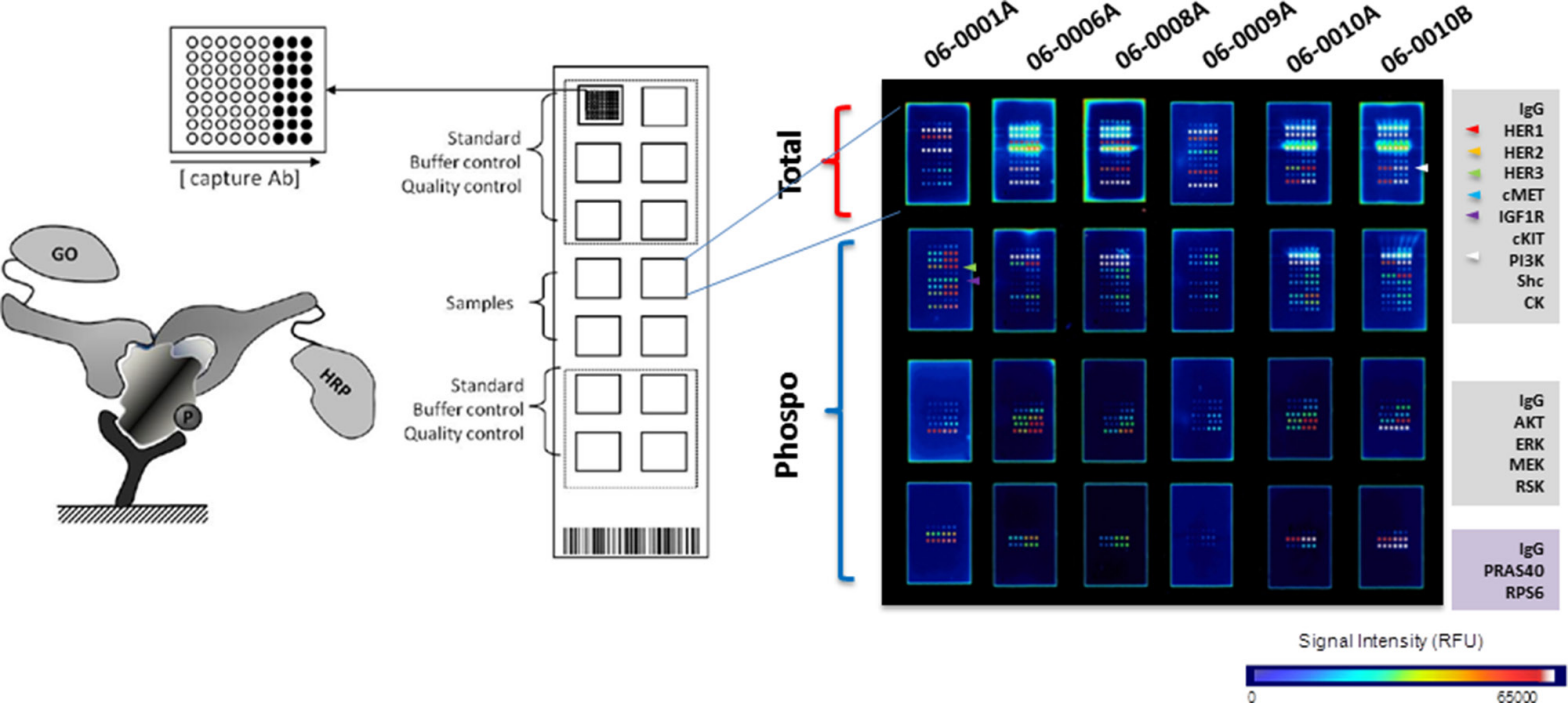
Expression and Phosphorylation of RTKs and downstream signaling molecules in NSCLC Immunoarray technology, Collaborative Enzyme Enhanced Reactive-immunoassay (CEER™), was utilized to determine the level of expression and degree of phosphorylation in tumor cells isolated from specimens collected from NSCLC patients. Schematic assay principle and assay format is shown on the left. Each array contains designated standards and controls; multiple photomultiplier (PMT) settings are utilized to have expanded dynamic range of signal quantitation and signals for clinical samples are reported after normalizing against standards on each slide. Capture antibodies printed on microarray surface in triplicate with two dilutions are indicated (right).

RTK profile analysis using collaborative enzyme enhanced reactive-immunoassay (CEER™) was performed on tumor specimens collected from 34 patients who received EGFR TKIs. RTKs were detected in 29 out of 34 samples (85.3%) and all 29 of these showed some degree of EGFR expression ranging from 0.5 CU to 193 CU (Supplementary Table S1). Examples of immunoarray expression and activation profiles are shown in Fig. 1. There was no significant difference between the levels of EGFR expression in *EGFR*-activating mutation positive patients vs. activating mutation negative patients with median expression of 66.3 CU and 55.9 CU respectively, *p* = 0.6906 (Supplementary Fig. S2).

**Figure 2:**
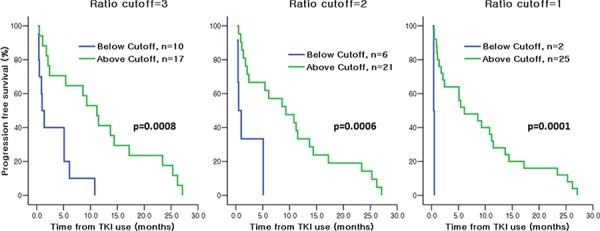
Kaplan-Meier analysis of PFS according to baseline EGFR/MET Index in NSCLC patients (*N* = 27, all genotypes included) treated with EGFR TKIs A striking separation of PFS was observed between NSCLC patients with high EGFR/MET relative ratio vs. low EGFR/MET relative ratio at multiple cut-offs.

**Figure 3:**
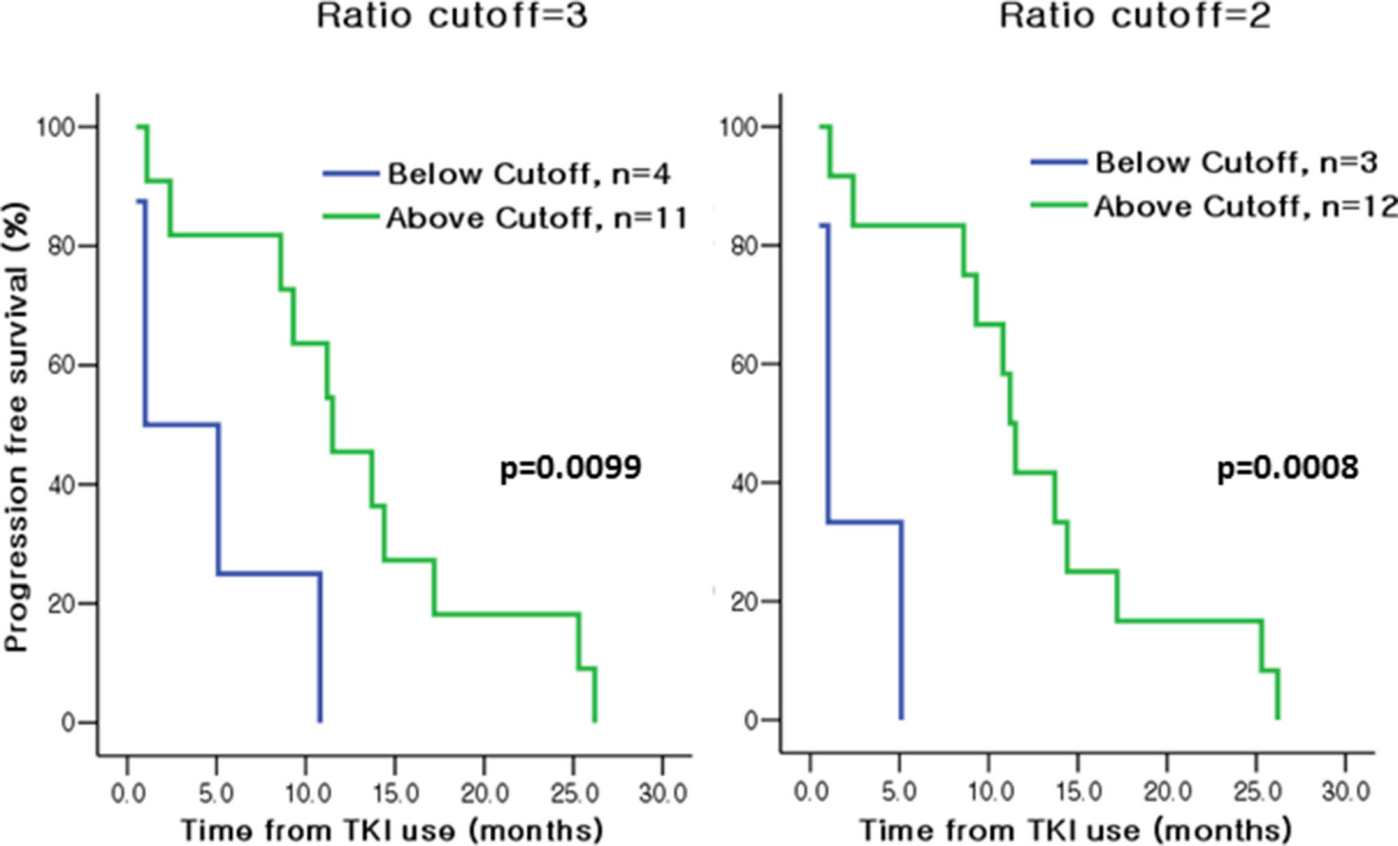
Kaplan-Meier analysis of PFS according to baseline EGFR/MET Index in NSCLC patients with EGFR-activating mutations (*N* = 15) treated with EGFR TKIs A striking separation of PFS was observed between NSCLC patients with high EGFR/MET relative ratio vs. low EGFR/MET relative ratio even among patients with *EGFR*-activating mutations.

Furthermore, levels of MET and HER3 were quantitated in the 34 baseline tumor specimens collected (Supplementary Table S1). While MET was widely expressed in 25 out of 29 RTK-positive samples (86.2%), HER3 expression was more limited in 24% of RTK-positive samples. When analyzing these RTK profiles, we focused on patients whose response was discrepant from general expectation (006-004, 006-032, 006-044). Remarkably, we observed that a patient with *EGFR* wild-type genotype who experienced a prolonged clinical response (006-004) exhibited high EGFR/MET ratio. On the contrary, two *EGFR*-mutant patients who did not respond to EGFR TKIs (006-032 and 006-044) had high levels of MET (or low EGFR/MET ratio) suggesting the EGFR axis may not be the main driver in these cases. Next, we evaluated the quantitative relationship between EGFR and MET in the entire cohort to assess whether any correlation with the clinical response to EGFR inhibitors existed. Fig. 2 depicts the Kaplan-Meier PFS plots according to EGFR/MET ratio for the 27 patients with evaluable information on both EGFR/MET ratio and PFS who received EGFR TKIs. Indeed, a striking difference in PFS was observed: regardless of *EGFR* mutation status, patients with high levels of EGFR relative to MET (or higher EGFR/MET ratios) experienced increased median PFS compared to those with lower EGFR/MET ratios, with statistical significance reached at multiple EGFR/MET ratio cut-offs (6.1 vs. 0.4 months, *p* = 0.0001 with ratio cut-off of 1; 9.3 vs. 0.5 months, *p* = 0.0006 with ratio cut-off of 2; 11.2 vs. 1.0 months, *p* = 0.0008 with ratio cut-off of 3, Fig. 2). Furthermore, despite bearing *EGFR*-activating mutations, NSCLC patients with increased MET expression relative to EGFR (or decreased EGFR/MET ratio) experienced a worse clinical outcome with short median PFS (1.0 vs. 11.2 months, *p* = 0.0008 with EGFR/MET ratio cut-off of 2; 1.0 vs. 11.5 months, *p* = 0.0099 with EGFR/MET ratio cut-off of 3, Fig. 3). These results suggest that, in this limited cohort, the EGFR/MET ratio measured in tumors at baseline is an effective stratifier of PFS in response to single agent EGFR TKIs, irrespective of patients’ *EGFR* genotype.

Of note, 7 out of 7 (100%) patients with PFS greater than 12 months showed no evidence of HER3 expression in their tumors, while 6 out of 20 (30%) patients with PFS lower than 12 months carried tumors positive for HER3 (Supplementary Table S2). We also analyzed the RTK profile of samples collected at different time-points throughout TKI therapy in 5 patients (Supplementary Table S3). A comprehensive expression and phosphorylation profile was observed in one clinical case (006-010): a reduction in EGFR, HER2 and HER3 phosphorylation was observed at the end of treatment compared to a pre-treatment sample most likely reflecting on-target inhibition of EGFR homo- and heterodimer signaling by gefitinib. However, upon disease progression on EGFR TKI therapy, a striking increase in MET activation was observed in tumor cells from patient 006-010, possibly reflecting a compensatory mechanism for tumor growth.

## DISCUSSION

In order to evaluate heterogeneity among NSCLC tumors, we quantitated the expression and phosphorylation levels of common RTKs, such as the HER family, MET and IGF-1R, in tumor cells isolated from pleural effusion and/or fine needle aspirations (FNAs). Biological specimens were collected from stages IIIB to IV NSCLC patients at baseline, prior to the start of EGFR TKIs. While 85% of tumors analyzed expressed at least one RTK, the vast majority expressed 3 or more RTKs, all potential molecular drivers of cell proliferation and tumor invasion. Thus, a comprehensive evaluation of growth signaling pathways in NSCLC tumors is essential in order to better strategize treatment options in the clinic.

In the present study, 56% of patients harbored activating *EGFR* mutations, while tumor EGFR expression was observed in 85% of all patients, regardless of their *EGFR* genotype. In the absence of an analytical tool to assess the functional status of EGFR, the activating mutations serve as the primary biomarkers for selecting NSCLC patients who will most likely benefit from EGFR TKIs. While this approach does help identify a substantial portion of responders, 30–40% of *EGFR*-mutant patients are primary non-responders to EGFR TKIs. Furthermore, many initial responders will eventually develop resistance to EGFR inhibitors. Hence, there is a critical unmet need for additional biomarker(s) of response to single agent EGFR inhibitors in order to improve current patient selection methods in NSCLC. Here, we report that combined analysis of RTK proteins, EGFR and MET, may be useful in identifying NSCLC patients most likely to experience a clinical response with prolonged PFS to EGFR TKIs. The current study suggests that higher EGFR/MET ratios correlate with a tumor's addiction to the EGFR pathway. It is expected that NSCLC patients with EGFR-driven disease will show a substantial PFS advantage over patients with both EGFR and MET-driven disease (as evidenced by lower EGFR/MET relative ratios; continuous variable) when treated with EGFR inhibitors. Indeed, over 10 months of PFS advantage was observed in patients with EGFR/MET relative ratios above 3 when compared with patients with EGFR/MET relative ratios below 3. Of note, patients with higher levels of EGFR to MET experienced increased PFS regardless of their *EGFR* mutation status. Hence, it is logical to speculate that the relative ratio of EGFR/MET may serve as a predictive biomarker for EGFR inhibitor treatment in NSCLC patients and warrants further investigation in an expanded prospective clinical trial. If validated, these results may open up additional treatment options to include EGFR TKIs for selected *EGFR* wild-type NSCLC patients.

The quantitative and comparative evaluations of EGFR and MET potentially identify a subset of patients who may benefit from dual blockade of these competing drivers in NSCLC tumors. The phase III randomized trial MARQUEE (NCT01244191) studied the dual blockade of EGFR and MET, with small molecule inhibitors Erlotinib and Tivantinib respectively, in stage IIIB/IV non-squamous NSCLC patients with 1 or 2 prior chemotherapies and no prior therapy with an EGFR or MET inhibitor [37]. This trial was performed in a broad un-selected non-squamous NSCLC population with 90% *EGFR* wild-type patients. The investigators, however, did incorporate a non-mandatory biomarker component evaluating MET expression by IHC, *MET* amplification by FISH and circulating hepatocyte growth factor levels (HGF, ligand for MET) for subgroup analyses. The trial was prematurely terminated after interim analysis reported that the study failed to meet its primary endpoint of an overall survival (OS) benefit in the experimental arm. In a similar study, the phase III MetLUNG trial investigated the impact on OS of adding onartuzumab, a monoclonal antibody against MET, to erlotinib in second or third-line therapy of advanced NSCLC patients with MET overexpression (NCT01456325). MET positivity was assessed centrally using IHC and only patients with scores of 2+ or 3+ were enrolled, while 89% of patients were *EGFR* wild-type. This study was also prematurely terminated as it did not achieve any improvement in OS or PFS in the combined arm [38]. Both of these failed studies, however, did not consider the relationship between EGFR and MET as patient selection criteria. Based on our findings, NSCLC patients with lower EGFR/MET relative ratios, possibly reflecting a requirement for both signaling pathways to sustain tumor growth, may be the ideal patient subgroup to benefit from combined EGFR and MET inhibition compared to single agent therapy. This hypothesis should be tested in future prospective clinical studies.

Tumors are known to evolve under therapeutic pressure, it is therefore extremely important to continuously monitor tumor growth signaling pathways to keep the disease under control. In this study, we observed an increase in activation of an alternate and compensatory RTK, MET, at the time of progression on EGFR inhibitor treatment. This compensatory signaling occurred despite initial tumor shrinkage and clinical response to gefitinib. Patients with detectable and activated alternate signaling in their tumors may potentially benefit from a combination of targeted therapies. Hence, in order to evaluate the functional status of multiple signaling proteins in biological specimens with very limited availability, a sensitive and specific platform is required. In this report, we have successfully demonstrated the use of a proximity triplex immunoarray technology for this purpose.

In conclusion, our results suggest the EGFR/MET ratio measured in tumors at baseline may help identify NSCLC patients most likely to benefit with prolonged PFS when treated with EGFR inhibitors. The current NSCLC patient selection method based on activating *EGFR* mutation status as primary biomarker of response to EGFR TKIs has proven clinical utility, but remains imperfect as it is unable to identify *de novo* non-responders. Based on this study, we hypothesize that comprehensive molecular profiling of multiple potentially competing RTKs can substantially improve probability of selecting responders to EGFR inhibitors. The expanded pathway-guided therapeutic strategies based on the relative ratios between different tumor RTK drivers should be further validated for clinical use.

## MATERIALS AND METHODS

### Patient cohort and tissue specimen procurement

This is a prospective pilot study conducted at Samsung Medical Center in South Korea, and patient recruitment occurred between May 2010 and Dec 2012. Patients were eligible if they had histologically or cytologically diagnosed advanced stage IIIB/IV NSCLC. Other inclusion criteria included age 18 years or older, an Eastern Cooperative Oncology Group (ECOG) performance status of 0–2, adequate organ function with estimated creatinine clearance ≥50 ml/min, and one or more measurable lesion. Exclusion criteria included uncontrolled diabetes mellitus, heart disease, obstructive pneumonia, infection, and uncontrolled symptomatic brain metastasis. Eligible patients received gefitinib (250 mg daily per oral), erlotinib (150 mg daily per oral) or afatinib (40 mg daily per oral) until disease progression or unacceptable adverse event. The study was performed in accordance with Good Clinical Practice guidelines and the Declaration of Helsinki. All patients provided written informed consent. This study was approved by an independent ethics committee.

Tumor specimens were collected either by isolating cells from pleural fluid or *via* fine needle aspiration (FNA) process from 37 NSCLC patients as summarized in Table 1. From each patient enrolled who met the inclusion criteria, a sample was taken before the beginning of EGFR TKI treatment. Whenever possible, FNA or pleural fluid specimens were collected 4 weeks after starting EGFR TKI therapy and at the end of treatment. Tumor cells present in pleural fluid were immediately centrifuged and the resulting cell pellet was resuspended in cell lysis buffer, while samples collected by FNA process were directly lyzed without centrifugation. The resulting lysates were shipped to Prometheus Laboratories (San Diego, CA) at ambient temperature within 48 hours of sample collection.

### Evaluation of clinical outcomes and statistics

Baseline tumor measurements were performed by computed tomography (CT) scan or magnetic resonance imaging (MRI) within 4 weeks of study entry. The response was assessed by CT scan every two cycles according to Response Evaluation Criteria In Solid Tumors (RECIST) version 1.1 [34]. Patients were categorized as responders to EGFR TKI when they met criteria for complete response (CR), partial response (PR), or stable disease (SD), whereas remaining patients with progressive disease (PD) were defined as non-responders. PFS is defined as the duration of time from start of treatment to time of progression, death from disease, the last follow-up or the starting date of salvage chemotherapy. For calculating and comparing PFS, the Kaplan-Meier method was used followed by the Log-rank test. Statistical analysis was performed using the Statistical package for the Social Sciences (SPSS) version 17.0 (SPSS Inc., Chicago, IL).

### Multiplexed-microarray printing

Capture antibodies (Abs) were printed on nitrocellulose-coated glass slides (ONCYTE®, Grace Bio-Labs) using a non-contact printer (Nanoplotter, GeSiM). The spot diameter was approximately 175 μm, slides were kept in a desiccated chamber. Approximately 500 pL of capture Abs were printed in triplicate and serial dilution concentrations of 1 mg/mL and 0.5 mg/mL. Purified mouse-IgGs served as negative controls. Immunoarray slide configurations and assay format was described previously [35].

### Collaborative enzyme enhanced reactive-immunoassay (CEER™)

Microarray based assays were performed as previously described [35]. Briefly, immunoarray slides were rinsed with TBST (50 mM Tris/150 mM NaCl/0.1% Tween-20, pH 7.2-7.4) and blocked for 1 hour at room temperature (RT). Serially diluted lysate controls in 80 μL dilution buffer (2% BSA/0.1% TritonX-100/TBS, pH 7.2-7.4) and samples were added to designated sub-arrays on slides, then incubated overnight at RT. After several washes, slides were incubated with two detector Abs (for different epitopes) conjugated with glucose oxidase (GO) and horseradish peroxidase (HRP) respectively for 2 hours at RT. After washing slides with TBST to remove unbound detector Abs, GO/HRP-mediated tyramide signal amplification process was triggered by adding biotin-tyramide solution and incubating for 30 mins. Local deposition of biotin-tyramide was detected by incubation with streptavidin-Alexa Fluor647 (Life Technologies, Carlsbad, CA) for 40 min. Slides were washed with TBST, dried and immediately processed on a high-resolution fluorescence microarray scanner (PowerScanner, Tecan).

For each marker, a sigmoidal standard curve was generated from eight concentrations of serially diluted lysates prepared from specific cell lines. HCC827, a NSCLC adenocarcinoma cell line carrying *EGFR* gene amplification and exon 19 deletion, was used for EGFR and MET quantifications, while T47D, a breast cancer cell line, served for HER2, HER3, IGF1R and PI3K quantifications. Alternatively, standard curves were generated from serially diluted recombinant proteins (AKT and ERK assays). Each curve was plotted as a function of signal intensity measured as relative fluorescence unit (RFU) vs. log concentration derived units, Computed Unit (CU). The data were fit to a five parameter equation by nonlinear regression, simultaneously fitting both dilutions of the capture Ab as described previously [35, 36]. CU is a representation of marker expression in unknown samples relative to that of control cell lines with known expression levels. Because expression of each marker is determined in unique CEER™ assays with different cell line standards, only CU values of the same marker across various samples can be compared.

### Mutation analysis

DNA from tumor tissue was extracted using the DNeasy Blood and Tissue Kit or the QIAamp DNA FFPE Tissue Kit (both from Qiagen, Hilden, Germany) according to the manufacturer's protocol. *EGFR* mutational analyses were performed by directional sequencing or PNA clamp method.

## SUPPLEMENTARY FIGURES AND TABLES


