# Supplementary material for: Low EGFR/MET ratio is associated with resistance to EGFR inhibitors in non-small cell lung cancer
Source: Oncotarget. 2015 Sep 3;6(31):30929–38. doi: 10.18632/oncotarget.5131 (PMC4741578; doi:10.18632/oncotarget.5131)
Supplement: Supplementary file 1 [file oncotarget-06-30929-s001.pdf]

SUPPLEMENTARY FIGURES AND TABLES

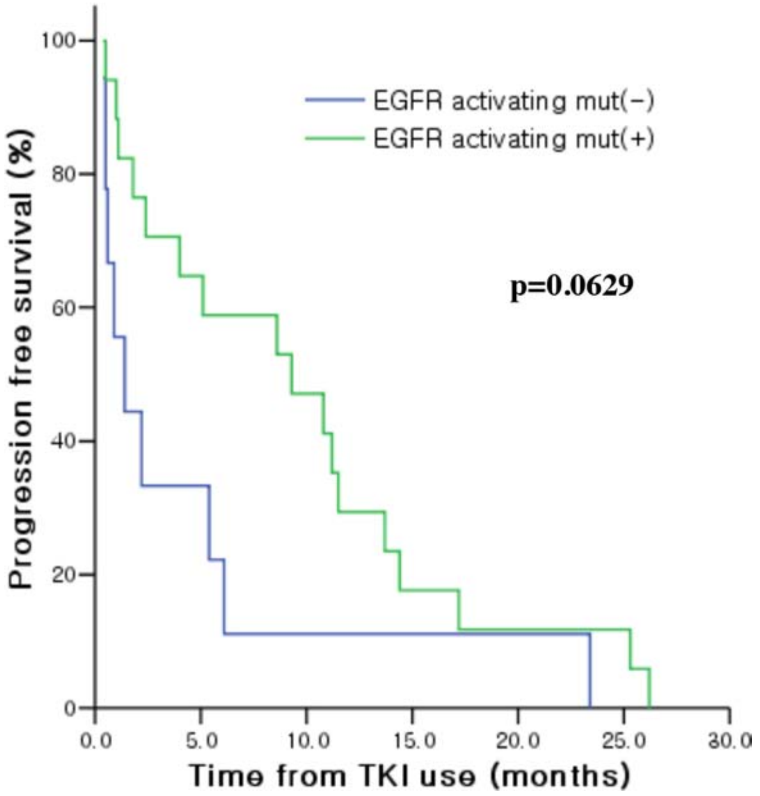

Supplementary Figure S1: Kaplan-Meier PFS analysis according to *EGFR* mutation status in NSCLC patients treated with EGFR TKIs. Out of an initial cohort of 34 NSCLC patients, 26 patients had evaluable PFS data and *EGFR* mutation information. The *EGFR* wild-type or non-activating mutation group (n = 9) had a median PFS of 1.4 months compared to 9.3 months for the *EGFR*-activating mutation positive group (n = 17). The PFS difference observed between the two groups did not reach statistical significance.

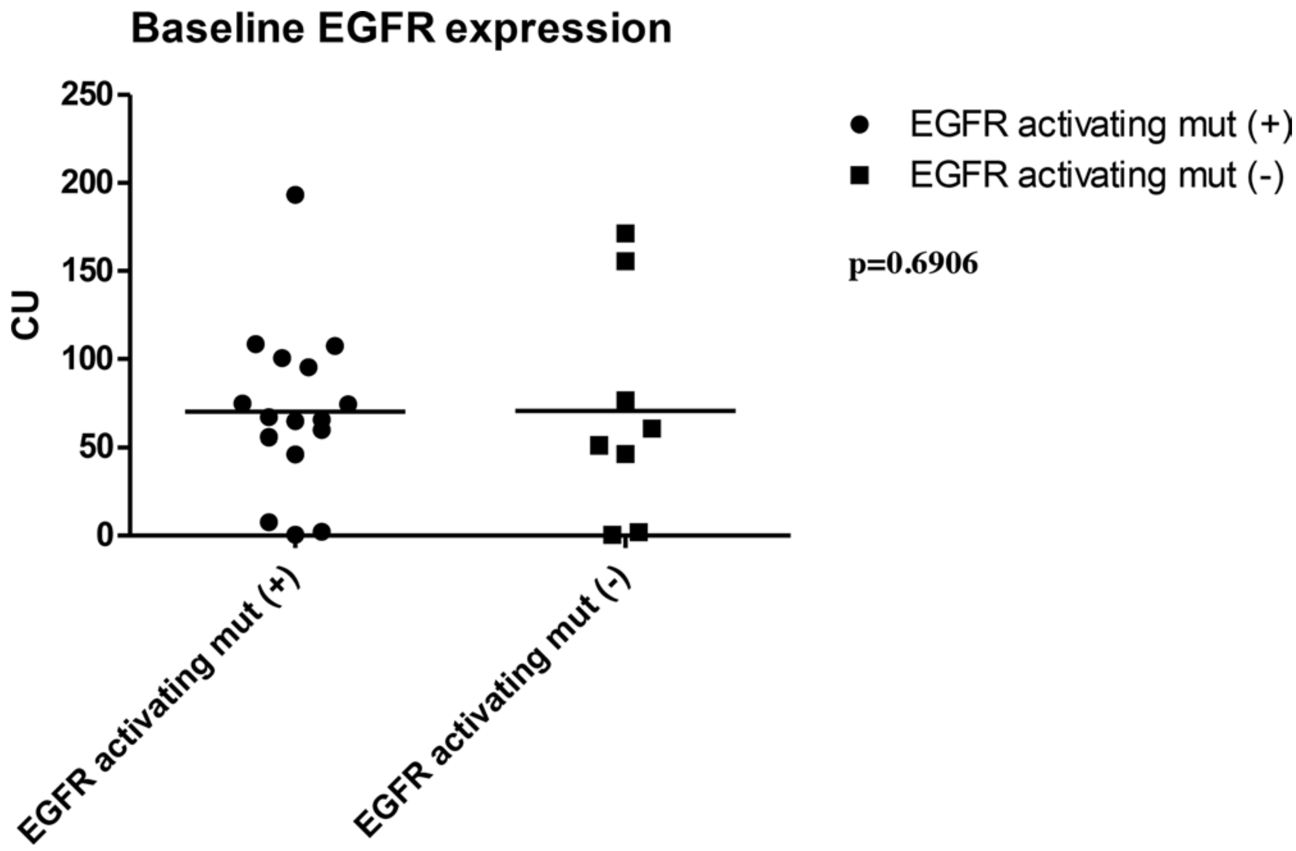

Supplementary Figure S1: Baseline EGFR expression according to *EGFR* mutation status in NSCLC patients. Out of an initial cohort of 34 NSCLC patients, 24 patients had quantifiable baseline EGFR levels by CEER™ and *EGFR* mutation information. The *EGFR*-activating mutation positive group (n = 16) had a median EGFR expression of 66.3 CU vs 55.9 CU for the *EGFR* wild-type or non-activating mutation group (n = 8). No significant difference was observed between the two groups.

Supplementary Table S1: Multiplexed RTK profile in NSCLC determined by CEER™

Patient Number	Sex	EGFR	MET	HER2	HER3	Patient Number	Sex	EGFR	MET	HER2	HER3
006-002	F	59.6	349.96	1108.77	_	006-026	F	107.46	546.47	1171.7	139.51
006-003	M	74.35	174.07	257.23	_	006-027	M	_	_	_	_
006-004	F	60.61	180.1	193.4	_	006-028	F	55.79	1432.94	1229.93	37.01
006-005	M	22.53	301.46	173.32	_	006-029	F	193.33	691.55	1781.91	27.67
006-006	F	171.33	1531.44	3115.19	306.21	006-030	F	0.97	_*	_	_
006-008	M	155.5	1234.39	4135.19	211.55	006-031	F	100.44	880.02	1062.08	_
006-009	M	22.08	36.38	209.8	_	006-032	M	95.4	H (>2000)	1215.65	80.9
006-010	M	170.91	H (>2000)	1616.76	65.54	006-034	F	_	_	_	_
006-012	F	2.01	_*	_	_	006-035	F	_	_	_	_
006-013	F	65.51	338.6	436.81	_	006-037	F	46.07	799.87	537.07	_
006-014	F	51.19	473.64	336.86	_	006-038	M	76.58	H (>2000)	852.68	_
006-017	M	109.36	304.62	313.77	_	006-039	F	46.35	1042.02	520.72	_
006-018	F	0.53	_*	_	_	006-040	M	67	197.25	298.4	_
006-019	M	_	_	_	_	006-041	F	7.69	27.59	106.45	_
006-021	F	0.46	_*	_	_	006-042	M	_	_	_	_
006-023	M	74.64	285.44	2634.46	_	006-043	M	2.28	8.28	70.93	_
006-025	F	64.59	319.32	641.69	_	006-044	M	108.43	1246.51	571.35	_

RTK expression was quantified in specimens collected from 34 patients treated with TKIs. RTK values shown are in Computed Unit (CU). RTKs are expressed in 29 out of 34 cases (85.3%).
'_' denotes an undetectable signal; 'H' denotes a signal above the upper limit of quantitation of the assay, in the case of MET H is > 2000 CU;
'_*' indicates the lower limit of detection of 2.5 CU for MET was used to determine EGFR/MET ratio.

Supplementary Table S2: HER3 expression in NSCLC specimens

Patient Number	*EGFR* activating mutation	TKIs	PFS with TKI	Best response to TKI	HER3	Patient Number	*EGFR* activating mutation	TKIs	PFS with TKI	Best responseto TKI	HER3
006-002	Positive	Gefitinib	**26.2**	PR	_	006-023	Positive	Gefitinib	**17.2**	PR	_
006-003	Positive	Erlotinib	1.1	SD	_	006-025	Positive	Erlotinib	**13.7**	PR	_
006-004	Negative	Gefitinib	**23.4**	PR	_	006-026	Positive	Gefitinib	11.2	PR	139.51
006-005	NA	Erlotinib	0.2	PD	_	006-029	Positive	Gefitinib	9.3	PR	27.67
006-006	Negative	Gefitinib	0.9	PD	306.21	006-030	NA	Gefitinib	2.1	SD	_
006-008	Negative	Erlotinib	6.1	PR	211.55	006-031	Positive	Erlotinib	10.8	PR	_
006-009	NA	Gefitinib	1.8	PD	_	006-032	Positive	Erlotinib	0.5	PD	80.9
006-010	NA	Gefitinib	5.1	PR	65.54	006-037	Positive	Gefitinib	5.1	PR	_
006-012	Negative	Gefitinib	5.4	SD	_	006-039	Negative	Gefitinib	0.4	SD	_
006-013	Positive	Gefitinib	8.6	PR	_	006-040	Positive	Erlotinib	**14.4**	PR	_
006-014	Negative	Gefitinib	1.4	PD	_	006-041	Positive	Gefitinib	2.4	SD	_
006-017	NA	Erlotinib	**27.1**	PR	_	006-043	Positive	Erlotinib	11.5	PR	_
006-018	Negative	Gefitinib	0.5	PD	_	006-044	Positive	Afatinib	1	PD	_
006-021	Positive	Gefitinib	**25.3**	PR	_						

Out of an initial cohort of 34 patients, 27 patients have evaluable progression free survival (PFS) data and an RTK profile. Among these, tumor HER3 expression was absent in 100% (7/7) patients with PFS ≥ 12 months (in bold), while 30% (6/20) patients with PFS < 12 months display tumor HER3 expression. HER3 values shown are in Computed Unit (CU).
PFS: progression free survival in months; PR: partial response; SD: stable disease; PD: progressive disease; NA: not assessed.

Supplementary Table S3: RTK profile analysis in NSCLC specimens taken from 5 patients at different time-points on EGFR TKI therapy

Patient ID	EGFR TKIs (Activating mutation)	T*	EGFR	HER2	HER3	MET	IGF-1R	PI3K	pEGFR	pHER2	pHER3	pMET	pIGF-1R	pPI3K	pSHC
**006-010**	Gefitinib (NA)	(1)	170,909,166	80,838,004	655,412	>100,000,000	1,037,498	5,608,644	5,621,454	1,063,121	198,166	35,392	31, 895	48,930	54, 022
		(3)	162,387,169	42,678,731	834,451	93,919,132	996,841	10,267,832	2,680,397	241,876	69,079	128,244	28,234	20,306	18,221
**006-012**	Gefitinib (Negative)	(1)	2,011,634	L	L	L	L	699,239	L	L	L	L	L	L	L
		(3)	1,006,310	L	L	L	L	L	L	L	L	L	L	L	L
**006-013**	Gefitinib (Positive)	(1)	65,505,447	21,840,491	L	16,930,088	L	1,114,338	L	L	L	L	L	L	L
		(2)	51,695,871	8,434,599	L	6,747,955	L	619,891	L	L	L	L	L	4,974	L
**006-014**	Gefitinib (Negative)	(1)	51,188,224	16,842,995	L	23,681,755	L	3,636,907	L	L	L	L	L	L	L
		(2)	51,725,694	15,787,747	L	19,365,391	L	1,150,462	L	L	L	L	L	4,733	L
		(3)	36,419,314	13,797,670	L	21,623,794	L	1,517,533	L	L	L	L	L	L	L
**006-037**	Gefitinib (Positive)	(1)	46,072,968	26,853,672	L	39,993,718	L	1,301,773	11,708	L	L	L	L	L	L
		(3)	3,137,553	1,448,861	L	5,857,209	L	L	L	L	L	L	L	L	L

* The RTK profile of samples collected at different time-points throughout treatment was determined by CEER™. A comprehensive expression and phosphorylation (p) profile was observed in one clinical case (006-010). Corresponding immunoarray is shown in Figure 1. Measured CU values are converted into relative number of marker expression or activation.
T represents timing of tissue acquisition: (1) pre-treatment; (2) 4 weeks after onset of EGFR TKI treatment; (3) end of treatment
NA: not assessed; L: marker levels below limit of detection.
